# Strong Symbiodiniaceae Influence on Coral Gene Expression Under Ocean Acidification and Warming

**DOI:** 10.1093/icb/icag062

**Published:** 2026-06-03

**Authors:** Colleen B Bove, Karl D Castillo, Annabel M Hughes, Justin B Ries, Sarah W Davies

**Affiliations:** Department of Biology, Ursinus College, Collegeville, PA, 19426, USA; The Department of Biology, Boston University, Boston, MA, 02215, USA; Department of Earth, Marine and Environmental Sciences, The University of North Carolina at Chapel Hill, Chapel Hill, NC, 27599, USA; Department of Earth, Marine and Environmental Sciences, The University of North Carolina at Chapel Hill, Chapel Hill, NC, 27599, USA; The Department of Biology, Boston University, Boston, MA, 02215, USA; Department of Marine and Environmental Sciences, Northeastern University, Nahant, MA, 01908, USA; Department of Marine and Environmental Sciences, Northeastern University, Nahant, MA, 01908, USA; The Department of Biology, Boston University, Boston, MA, 02215, USA

## Abstract

Tropical coral reefs face unprecedented threats from ocean acidification and warming, driving alarming declines in reef communities worldwide. Yet environmental history and diverse symbiotic partnerships often shape how corals respond to environmental change. We investigated how the Caribbean coral *Siderastrea siderea* responds to simulated future ocean conditions by examining holobiont phenotypes, symbiotic communities, and gene expression profiles. After three months of exposure to various acidification and warming scenarios, *S. siderea* showed only moderate stress responses, with no shifts in algal symbiont or bacterial communities. Remarkably, even under the warmest temperature and lowest pH conditions, coral host gene expression patterns were primarily shaped by which Symbiodiniaceae genus they hosted, rather than experimental treatments. Corals predominantly hosting *Durusdinium trenchii* exhibited higher lipid content but reduced calcification rates compared to those hosting *Cladocopium goreaui*, suggesting different metabolic strategies based on which symbiont was predominant in the coral holobiont. While moderate treatment effects were observed, significant changes in holobiont phenotype and gene expression occurred mainly under extreme acidification conditions unlikely to be experienced within the next century. Under these extreme scenarios, we detected reduced growth rates and downregulation of calcification-related genes, indicating potential challenges for skeletal production in future oceans. These findings enhance our understanding of coral acclimatization strategies and emphasize how symbiotic relationships fundamentally shape coral responses to environmental change. As climate change intensifies, these molecular and physiological mechanisms may determine which coral species persist on future reefs.

## Introduction

The partial pressure of carbon dioxide in the atmosphere continues to increase at unprecedented rates due to human activities. As a result, the world’s oceans are warming (Tanaka and Houtan 2022) and seawater carbonate chemistry is shifting (Caldeira and Wickett 2003) in ways that alter the physiology of many marine organisms (Kroeker et al. 2013; Agostini et al. 2021; Vargas et al. 2022), especially foundation species that provide critical habitat. Tropical coral reefs are particularly vulnerable to these stressors because they already exist near their maximum thermal limit (Jokiel and Coles 1977) in oceans that have been warming for over a century (Bove et al. 2022c). Further, reef-building corals rely on elevated concentrations of bioavailable carbonate ions to produce new skeletal material, a process that is at risk due to reduced carbonate ion availability and higher proton (H^+^) concentrations (Kleypas et al. 1999). These environmental changes force corals to acclimate and/or adapt to their new conditions, or face mortality and ultimately extinction risk (Seebacher et al. 2015; Habary et al. 2017; Keshavmurthy et al. 2021).

To better understand the potential for adaptation and acclimation under global change, many studies have assessed the physiological effects of ocean acidification and warming on tropical corals. However, these studies suggest that responses will be highly variable across individuals, populations, and species (Bahr et al. 2018; Kornder et al. 2018; Bove et al. 2019). For example, previous work has reported negative (Okazaki et al. 2017; Anderson et al. 2019), neutral (Comeau et al. 2013; Bove et al. 2019), and even moderately positive (Okazaki et al. 2017) skeletal growth responses of corals under projected ocean acidification and warming conditions. Many mechanisms have been proposed to explain these variable growth responses observed, including environmental history and population-level resistance (Kenkel et al. 2015), differences in coral energy reserves (Anthony et al. 2009; Anderson et al. 2019), differences in ability to regulate calcifying fluid chemistry in support of calcification (Ries 2011; Ross et al. 2022), and divergent molecular processes that support basic life functions of corals (Kaniewska et al. 2012, 2015; Moya et al. 2012). Indeed, corals may divert cellular energy toward processes that promote survival and recovery post-stress events, such as increased respiration and metabolism, potentially at the expense of other physiological processes (Carreiro-Silva et al. 2014; Davies et al. 2016; Maor-Landaw and Levy 2016; Jiang et al. 2022). Understanding the molecular mechanisms underpinning coral phenotypic responses under projected global change scenarios may provide valuable insights into why such variation exists between individuals and species.

As global change continues to affect ocean conditions, coral symbioses are also in flux (Bourne et al. 2016), highlighting the need to understand how global change influences the coral holobiont (animal host, Symbiodiniaceae symbionts, bacteria, viruses, endolithic algae, etc.). Tropical corals host different species of algal symbionts that can ultimately alter the coral’s physiology through varying levels of stress tolerance and energy allocation to the host (Barfield et al. 2018; Cunning and Baker 2020; Allen-Waller and Barott 2023). For example, corals hosting the thermally tolerant algal symbiont *Durusdinium trenchii* have been found to exhibit lower growth rates (Pettay et al. 2015) and lower autotrophically derived carbon (Wall et al. 2020) than counterparts hosting other, less thermally tolerant algal symbionts. Along with Symbiodiniaceae, other members of the microbiome (i.e., bacteria, viruses, fungi) contribute to the health of the coral holobiont through essential nutrient cycling and transport (Bourne et al. 2016). However, environmental change is driving shifts in these microbial communities (Savary et al. 2021). While the ability of the coral host to restructure its microbial community composition in response to changes may ultimately predict the success of the individual (Voolstra and Ziegler 2020), not all corals exhibit this flexibility (Ziegler et al. 2019). Previous work on *S. siderea* on the Mesoamerican Barrier Reef and in Florida have shown that both algal and microbiome communities are shaped by reef environments (Baumann et al. 2018; Bonthond et al. 2018; Speare et al. 2020), but few have explored how these communities shift in response to stress. Thus, understanding the partnerships within the coral holobiont is critical for assessing coral phenotypes expressed under global change scenarios to better predict species and population-level responses.

Our previous work demonstrates that the Caribbean coral *S. siderea* can maintain net positive skeletal growth even under extreme ocean acidification and moderate warming conditions (Castillo et al. 2014; Bove et al. 2019; Aichelman et al. 2021), highlighting its resistance to global change stressors. In contrast, both host and algal symbiont physiology (i.e., energy reserves, chlorophyll a content, symbiont density) appear less resistant under ocean acidification, with measurable declines observed over the course of exposure (Aichelman et al. 2021; Bove et al. 2022a). Importantly, these physiological responses differed between inshore and offshore populations (Baumann et al. 2021; Bove et al. 2022a). However, the molecular mechanisms underlying the phenotypes observed in *S. siderea* under chronic experimental ocean acidification and warming conditions remain unclear. Here, we integrate the previously published coral holobiont physiology with new TagSeq gene expression profiling and ITS2/16S rRNA amplicon sequencing following the 3-month mesocosm experiment. Our results reveal the robust nature of *S. siderea* under moderate global change conditions and that differing algal symbiont communities ultimately predict coral host gene expression responses and some phenotypes.

## Materials and methods

### Experimental design

**Fig. 1:**
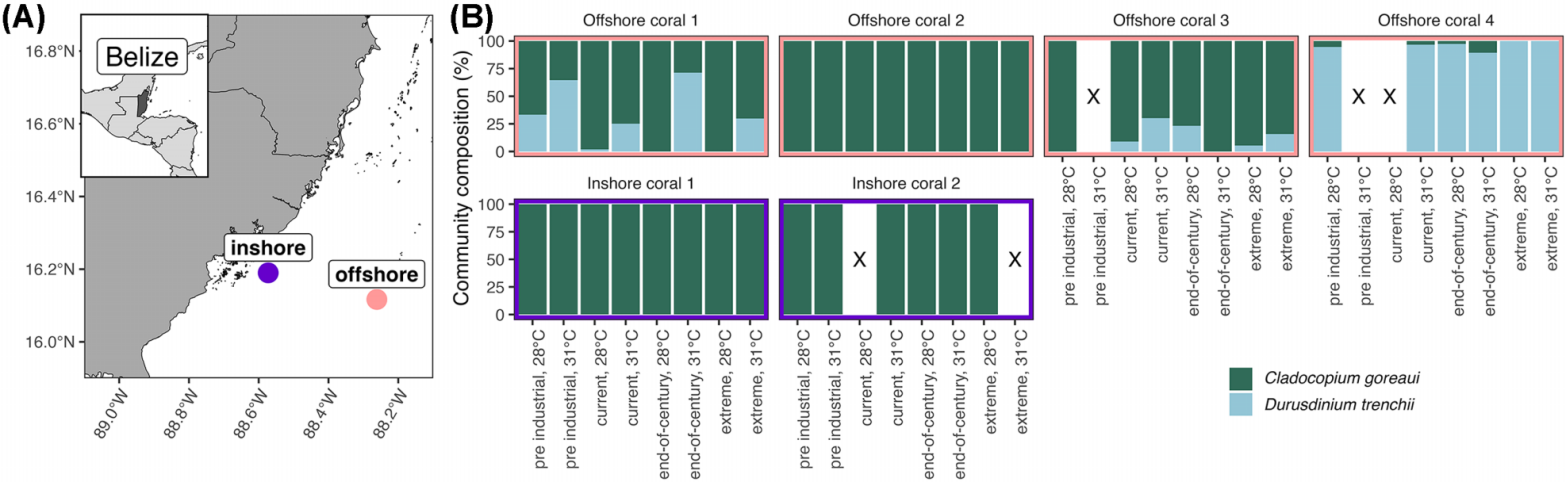
(**A**) Map of coral collection sites off the southern coast of Belize. The inshore site is located within the Port Honduras Marine Reserve (PHMR; purple) and the offshore location is within the Sapodilla Cayes Marine Reserve (SCMR; pink). Belize is highlighted in grey in the regional map insert for reference. (**B**) Relative proportion of algal symbiont ITS2 reads matching either *Cladocopium goreaui* (green) or *D. trenchii* (light blue) in experimental *S. siderea* fragments after 93 days of exposure to variable *p*CO_2_ (preindustrial, current, end-of-century, extreme) and temperature (28°C, 31°C). Each column represents a single coral fragment and the fragments within a box originate from the same colony. Corals collected from the offshore are depicted by pink boxes and inshore colonies are denoted by purple boxes. Columns with “X” represent samples that were removed due to low-quality ITS2 sequence data.

This study investigates the gene expression responses of the coral *S. siderea* that were previously assessed for calcification, linear extension, and physiology in Bove et al. (2019, 2022). *S. siderea* corals were collected from inshore (Port Honduras Marine Reserve; 16°11′23.5314″N, 88°34′21.9360″W) and offshore (Sapodilla Cayes Marine Reserve; 16°07′00.0114″N, 88°15′41.1834″W) reef environments from the southern portion of the Belize Mesoamerican Barrier Reef System in June 2015 (Fig. 1A). Corals were immediately transported to Northeastern University’s Marine Science Center and sectioned into eight equally-sized fragments that were randomly assigned to one of eight experimental treatments (three replicate tanks per treatment) for 93 days. The eight treatments encompassed four *p*CO_2_ treatments, which were achieved by bubbling either CO_2_-free air mixed with CO_2_ or air mixed with CO_2_ into each replicate tank, corresponding to pre-industrial, present-day, moderate end-of-century, and extreme (year 2500) *p*CO_2_, all crossed with two temperatures corresponding to the corals’ approximate present day summer mean (28°C) and projected end-of-century summer mean (31°C). These temperature-*p*CO_2_ (±SD) combinations resulted in eight triplicate treatments (24 tanks total): preindustrial (31.0 ± 0.04°C/288 ± 12 µatm *p*CO_2_; 27.9 ± 0.04°C/311 ± 18 µatm *p*CO_2_), present-day (28 ± 0.04°C/405 ± 17 µatm *p*CO_2_; 31.1 ± 0.05°C/447 ± 28 µatm *p*CO_2_), end-of-century (28.1 ± 0.05°C/701 ± 17 µatm *p*CO_2_; 30.9 ± 0.03°C/673 ± 19 µatm *p*CO_2_), and an extreme (28.1 ± 0.02°C/3309 ± 76 µatm *p*CO_2_; 31.0 ± 0.05°C/3285 ± 99 µatm *p*CO_2_) *p*CO_2_.

Corals were maintained in experimental aquaria filled with 5 μm-filtered natural seawater from Massachusetts Bay (salinity 31.7 [±0.2 SD]) and illuminated with full spectrum LED lights on a 10:14 light-dark cycle (PAR ca. 300 µmol photons m^–2^ s^–1^). Temperature, salinity, and pH were measured every other day and total alkalinity (TA) and dissolved inorganic carbon (DIC) were analysed every 10 days with a VINDTA 3C (Marianda Corporation, Kiel, Germany). Temperature, salinity, TA, and DIC were used to calculate carbonate parameters using CO_2_SYS (Pierrot et al. 2006) with Roy *et al*. (1993) carbonic acid constants K_1_ and K_2_, Mucci’s value for the stoichiometric aragonite solubility product (1983 ), and an atmospheric pressure of 1.015 atm. Further details on how salinity, temperature, pH, DIC, and TA were measured can be found in Bove et al. (2019). At the completion of the experimental period, corals were immediately flash-frozen in liquid nitrogen and transported to Boston University for downstream analyses. A subset of 43 *S. siderea* fragments from the original experiment was selected for gene expression profiling and metabarcoding of algal symbiont and bacterial communities at the completion of the experiment (Bove et al. 2019; Bove et al. 2022a). Fragment prioritization was based on representation from as many treatments as possible (*N* = 4–6 fragments per treatment) with colonies collected from both reef environments (*N*_offshore_ = 4 colonies; *N*_inshore_ = 2 colonies) to better understand how corals from different sites respond to independent and combined ocean acidification and warming at the gene expression level.

### ITS2 algal symbiont and 16S bacterial metabarcoding

Holobiont DNA was extracted using a phenol–chloroform extraction (Davies et al. 2013). Algal symbiont libraries were prepped by targeting the ITS2 region using *SYM_VAR_5.8S2* and *SYM_VAR_REV* primers (Hume et al. 2013, 2015) and the V4 region of the 16S rRNA gene was targeted with 16S_515F and 16S_806R primers with Illumina sequencing adaptors (Elmer et al. 2017) for bacterial community composition following protocols outlined in Bove et al. (2023). Concentrations of the ITS2 and 16S pools were used to combine the two pools in a 1:3 ratio, respectively, and were sequenced on a single lane of Illumina MiSeq (paired-end 250 bp) at Tufts Genomics Core Facility.

Demultiplexed reads were preprocessed using *bbmap* (Bushnell 2014) to separate ITS2 and 16S reads and to remove reads that did not include either primer. ITS2 reads were submitted to *SymPortal*, which identifies sets of defining intragenomic sequence variants (DIVs) to identify ITS2 type profiles that are indicative of genetically differentiated Symbiodiniaceae taxa (Hume et al. 2019). *SymPortal* identified that coral fragments hosted either *Cladocopium goreaui, D. trenchii*, or a combination of these two species based on type profiles (Fig. S1A), which informed downstream phenomic and gene expression analyses. While it is challenging to determine quantitative Symbiodiniaceae community composition due to ITS2 copy number variation (Davies et al. 2023), for this study, corals with less than 98.2% of Symbiodiniaceae-specific ITS2 reads assigned to *C. goreaui* were considered to be *D. trenchii* hosts based on overall shifts in host gene expression.

16S primers were removed from bacteria sequences using *cutadapt* (Martin 2011). DADA2 (Callahan et al. 2016) quality filtering was conducted and 10,466 sequence variants (ASVs) were inferred, and taxonomy was assigned at 100% sequence identity using the *Silva* v. 138.1 database (Quast et al. 2013). ASVs matching mitochondria, chloroplasts, or nonbacterial kingdoms were removed (765 total) and 15 ASVs were removed based on negative controls as contaminants (*decontam*; Davis et al. 2018). Cleaned counts were rarefied to 3729 using *vegan* (Oksanen et al. 2020) and resulting counts were trimmed using *MCMC.OTU* (Matz 2016) to remove ASVs with less than 0.01% of counts, resulting in 1023 ASVs across samples.

### Coral holobiont phenome

Physiological parameters, including coral calcification rate, host energy reserves (total protein, lipid, carbohydrate), algal symbiont density, chlorophyll a concentration, and coral tissue color intensity, of the fragments selected for this gene expression study were previously published (Bove et al. 2022a). However, these phenotypic traits (referred to hereafter as the “holobiont phenome”) provide important context for the gene expression responses of corals exposed to global change stressors, so these data were included in the present study to disentangle functional differences across treatments. Briefly, coral calcification rate was estimated using the buoyant weight technique (Spencer Davies 1989) from weights collected at the start and end of the 93-day experiment for each fragment. Images of each fragment were taken at each timepoint to calculate surface area. Color intensity was measured from these images using a custom Python script (Bove et al. 2022a) and the MATLAB (Winters et al. 2009) macro “AnalyzeIntensity” for 10 points on each fragment.

At the end of the experiment, coral tissue was removed from skeletons via airbrushing with saltwater (salinity 35) and then homogenized with a *Tissue-Tearor*. An aliquot of tissue slurry was taken for algal symbiont counts, which were conducted in triplicate 10 μL aliquots using a hemocytometer and standardized to total tissue volume and coral surface area. The tissue slurry was then centrifuged to separate the coral host tissue from the algal symbiont cells. Chlorophyll a was extracted from the algal pellet by adding 90% acetone to the sample at −20°C for 24 hours then quantified using a Turner Design 10-AU fluorometer with the acidification method (Parsons et al. 1984) to determine sample concentration per surface area. Coral host energy reserves (protein, carbohydrate, and lipid) were quantified using the host slurry after pelleting out the algal cells. Total protein was quantified using a Bradford assay and read at 562 nm on a spectrophotometer in duplicate. Host carbohydrate was measured using a modified phenol-sulphuric acid method suitable for 96-well plates (Masuko et al. 2005; Bove and Baumann 2021a) and read on a spectrophotometer at 485 nm. Coral host lipids were extracted following the Folch Method (Folch et al. 1957), assayed using a modified colorimetric 96-well plate protocol, and read on a spectrophotometer at 540 nm (Bove and Baumann 2021b; Cheng et al. 2011).

### Coral RNA isolation and gene expression profiling

Total RNA was isolated using the RNAqueous-Micro Total RNA Isolation Kit (*Invitrogen*) following the manufacturer’s protocols with one additional step. Prior to RNA isolation, samples were thawed on ice and placed in a bead beater for 1 min with glass beads and 150 μL of lysis buffer before proceeding with the manufacturer’s protocol. Trace DNA contamination was eliminated with DNase I (*Invitrogen*) digestion for 20 min at 37°C. Total RNA was transcribed into first-strand cDNA and resulting complementary DNA was prepared for TagSeq whole-genome gene expression profiling (Meyer et al. 2011). Each library received an individual barcode adapter through a secondary PCR and the pooled library of 43 samples was sequenced across two lanes of Illumina HiSeq 2500 at Tufts Genomics, yielding pair-ended (PE) 50 base pair (bp) reads. Raw reads for all samples are available on the NCBI Sequence Read Archive (SRA) under BioProject number PRJNA1465334.

Individual raw reads for each of the 43 libraries ranged from 16,502 to 13,562,193. Two samples (CNSD2 and CNSD8; Table S1) had low sequencing depth and were removed, resulting in an average of 7 million raw reads across the remaining 41 samples (range: 2–13 million; standard deviation [SD]: 2 million raw reads). Illumina adapters and poly(A) tails were removed from samples using Fastx_toolkit (http://hannonlab.cshl.edu/fastx_toolkit), sequences less than 20 bp in length with < 90% of bases having quality cutoff scores > 20 were trimmed, and PCR duplicates were identified and removed from all libraries. PCR duplicates can be removed because degenerate bases are incorporated during cDNA synthesis, allowing for PCR duplicate removal (Lohman, Weber and Bolnick 2016). Resulting trimmed reads (mean 1.3 ± 0.4 million SD per sample) were mapped to a holobiont transcriptome (*S. siderea* host [Davies et al. 2016] + *C. goreaui* [Davies et al. 2018] + *D. trenchii* [Shoguchi et al. 2021]) using Bowtie2.2.0 (Langmead and Salzberg 2012). All versions of read mapping references and associated annotation files used in this study have been included on GitHub (https://github.com/seabove7/SSID-gene-expression) and Zenodo (https://doi.org/10.5281/zenodo.20448169). Because samples hosted mixed algal symbiont communities, only gene expression data uniquely mapping to the *S. siderea* reference were assessed in depth. However, holobiont and *C. goreaui*-only data were explored for basic patterns of expression, which are described in more detail below.

Coral host mapped reads ranged from 50,888 to 783,503 counts across samples (mean 366,033 ± 166,454 SD per sample), accounting for 58–92% of the total holobiont reads (Table S1). Raw counts were *vst*-transformed (variance stabilizing transformation) and normalized for size factor differences using the median ratio method.

### Phenomic and gene expression statistical analyses

Differences in bacterial community composition across samples were assessed via alpha diversity (rarefied; Shannon index, Simpson’s index, ASV richness, and evenness) based on treatment using *Phyloseq* (function *estimate_richness*; McMurdie and Holmes 2013) and beta diversity (not rarefied) using a PCoA on Bray–Curtis dissimilarity with a PERMANOVA (*vegan* package, version 2.5.7 [Oksanen et al. 2020]). Alpha diversity metrics were assessed using additive linear mixed effects models (package *lme4*, version 1.1–28 [Bates and Maechler 2015]) with Symbiodiniacaeae genus, reef environment, and treatment as the predictors and a random effect of coral colony. The effect of experimental treatment on beta diversity was assessed using *betadisper* (*vegan* package; version 2.5.7 [Oksanen et al. 2020]). Finally, differentially abundant ASVs based on temperature treatment, *p*CO_2_ treatment, and dominant symbiont type were identified using an Analysis of Compositions of Microbiomes with Bias Correction (ANCOM-BC) (Lin and Peddada 2023). The false discovery rate was controlled using the Benjamini–Hochberg method and ASVs with *q*-value < 0.05 were considered significant. ANCOM analyses were performed at both the phylum and class levels.

Principal component analysis (PCA) (function *prcomp*) of scaled and centered holobiont phenome parameters were employed to assess the relationships between holobiont phenome, hosted Symbiodiniaceae genus, and experimental treatment conditions. Main effects (Symbiodiniaceae genus, temperature, *p*CO_2_, and reef environment) were evaluated using a PERMANOVA with the *adonis2* function (1500 iterations and euclidean distances; *vegan* package, version 2.5.7 [Oksanen et al. 2020]).

Significantly differentially expressed genes (DEGs) were identified by an FDR adjusted *P*-value < 0.1 in *DESeq2* (version 1.26.0; Love et al. 2014), with the model: *design* = ~ *colony* + *treatment*. A PCA was employed to test for global gene expression differences across treatments, reef environment, colony, and Symbiodiniaceae genus (function modified from *plotPCA*; package *DESeq2* [version 1.26.0; Love et al. 2014]) and assessed using the *adonis2* function.

To explore whether the patterns of gene expression of both the holobiont (*S. siderea* + *C. goreaui + D. trenchi*) and *C. goreaui* only were consistent with those patterns of the host, we conducted PCAs of *vst-*normalized gene expression of *C. goreaui* only and holobiont reads following methods described above. We also assessed numbers of DEGs in both data types. However, because these coral fragments host a mix of two symbiont genera, many TagSeq reads are likely to map equally well to highly conserved genic regions in *Cladocopium* and *Durusdinum*. In addition, Davies et al. (2018) previously found constitutive gene expression differences between *C. goreaui* associated with corals from different reef types, regardless of experimental conditions, further confounding our ability to explore symbiont gene expression. Therefore, because patterns of holobiont and *C. goreaui* expression were highly similar to those observed for the coral host, no further analyses were performed on the holobiont or *C. goreaui*-only datasets.

To further assess the effects of Symbiodiniaceae genus and *p*CO_2_ treatment on host gene expression, we used a discriminant analysis of principal components (DAPC) (package *adegenet*; version 2.1.4 [Jombart and Ahmed 2011]). Cross-validation was performed using the function *xvalDapc* to identify the optimum number of PCs to retain in each DAPC model (10 PCs for symbiont genera and 20 PCs for *p*CO_2_ treatment). All discriminant functions were retained for both DAPC models.

All statistical analyses and figure constructions were carried out in R version 4.6.0 (R Core Team 2020). The accompanying data and code can be freely accessed on GitHub (https://github.com/seabove7/SSID-gene-expression) and Zenodo (https://doi.org/10.5281/zenodo.20448169).

### Weighted gene correlation network analysis

To better link phenotypes with gene expression data, correlation structure within the transformed gene expression and phenotypic data was examined using a Weighted Gene Co-expression Network Analysis (WGCNA) to identify groups of genes (“modules”) that are co-regulated across *S. siderea* samples (*WGCNA* package; version 1.71 [Langfelder and Horvath 2008, 2012]). Expression of genes within each module is summarized by the overall expression and represented by the eigengene, which can then be correlated post hoc with phenotypic traits. Here, the traits considered were: experimental treatment, reef environment, Symbiodiniaceae genus, bacterial community diversity (evenness and PCoA axis 1), and all host and algal symbiont phenotypes. Resulting correlations were assessed to identify modules that capture eigengene expression that correlated with treatment responses and other physiological traits using a custom R function (github.com/seabove7/RandomFun/tree/main/WGCNA_auto).

Gene Ontology (GO) enrichment analysis was then applied to WGCNA modules using the GO_MWU package (flag “isModule = TRUE”) to identify enrichment categories within each selected module using Fisher’s exact tests that apply a statistically conservative presence/absence measure of a gene within a given module (Wright et al. 2015). Resulting significant GO terms were plotted as dendrograms that show the level of gene sharing across GO terms.

## Results

### Symbiodiniaceae community characterization and classification of coral fragments

ITS2 reads after submission to *SymPortal* averaged 17,988 ± 6,840 SD per sample, with a minimum of 5,676 counts (sample CFSF16) and a maximum of 42,779 counts (sample CFSA6). At the end of the experiment, all *S. siderea* coral fragments hosted only *C. goreaui, D. trenchii*, or a combination of these two algal symbiont genera from 12 different ITS2 type profiles (Figs. 1B and S1A). The two coral colonies collected from the inshore reef environment along with a single offshore coral individual (offshore coral 2) exclusively hosted *C. goreaui* algal symbionts regardless of experimental treatment. The other three offshore coral colonies associated with a mix of algal symbiont genera depending on individual fragments. Based on these results and patterns in the gene expression presented below suggesting that small percentages of *D. trenchii* modulate host gene expression, we classified coral fragments as either *C. goreaui* (> 98.2% reads assigned to *C. goreaui*) or *D. trenchii* (< 98.2% reads assigned to *C. goreaui*) hosting for downstream analyses to assess how symbiont community composition alters the coral phenome and host gene expression.

### Bacterial community composition did not differ across samples or treatments

**Fig. 2:**
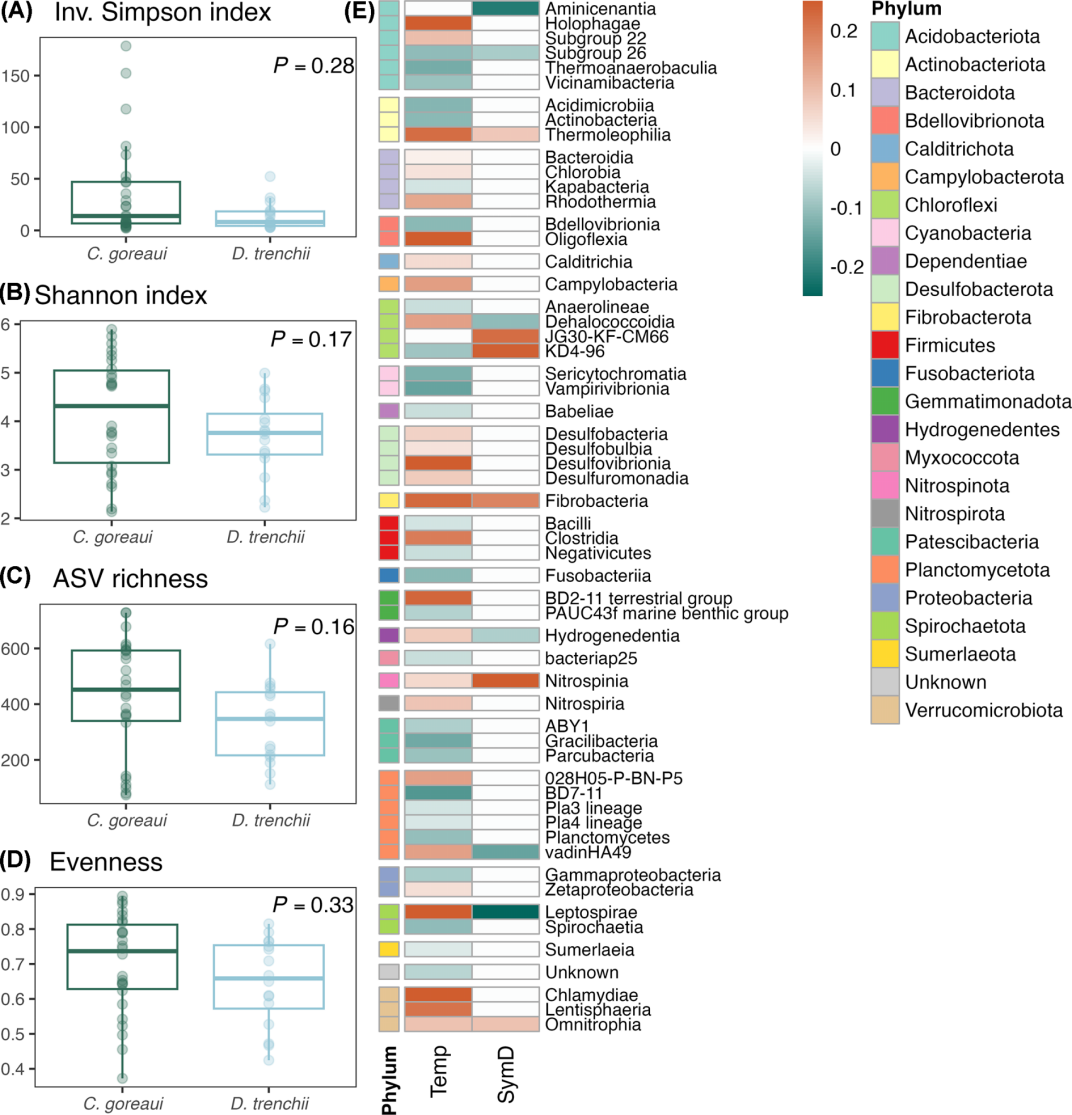
Mean coral-associated bacterial community alpha diversity (**A-D**) in hosts dominated by different symbiont genera. No differences were detected in any of the metrics evaluated [inverse Simpson index (**A**), Shannon index (**B**), ASV richness (**C**), and Evenness (**D**)]. Differentially abundant bacteria classes grouped by phylum based on temperature treatment (28°C vs. 31°C) or dominant symbiont genera hosted (SymD; *D. trenchii* vs. *Cladocopium goreaui*) as identified by ANCOMBC (**E**). Heatmap colors represent log foldchange of taxa abundance relative to all samples: red indicates increased abundance while green represents reduced abundance.

Cleaned 16S reads before trimming and rarefaction averaged 31,510 ± 16,865 SD per sample, with a minimum of 3,729 counts (sample CFSF5) and a maximum of 81,686 counts (sample CFSD7). The top five most common ASVs included four from the phylum Proteobacteria, including the genera MD3-55 (*Ca*. Aquarickettsia; ASV 1), Marine Methylotrophic Group 3 (ASV 6), Ruegeria (ASV 11), and Halioglobus (ASV 13), and a single ASV from the phylum Bacteroidota (Family Flavobacteriaceae; ASV 8) (Fig. S1B). Alpha diversity (Simpson index, Shannon index, ASV richness, evenness) was indistinguishable across all samples, regardless of treatment, Symbiodiniaceae genus, or reef environment (*P* > 0.05; Fig. 2A–D). Similarly, beta diversity analyses did not identify any statistical differences across samples based on experimental treatment, Symbiodiniaceae genus, or reef environment (*P* > 0.05; Fig. S1C).

Differential abundance analysis identified 55 bacterial classes with significant differences in relative abundance in response to temperature treatment from over 20 phyla (Fig. 2E). Only 12 bacterial classes were significantly differentially abundant between corals hosting *C. goreaui* versus *D. trenchii*, including Leptospirae and Nitrospinia (Fig. 2E). No differentially abundant classes were identified in response to ocean acidification treatments.

### Ocean acidification and warming alter coral holobiont phenotype

**Fig. 3:**
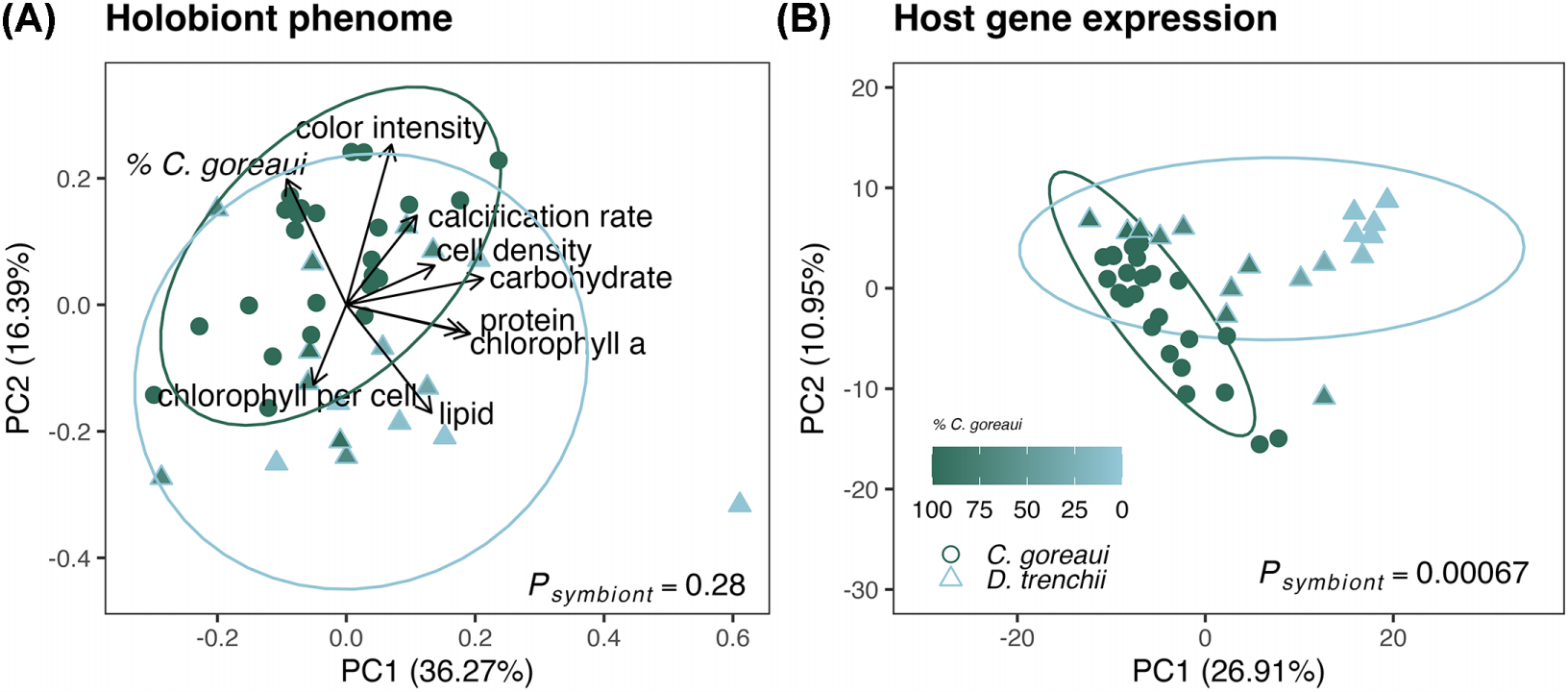
PCA of (**A**) *S. siderea* holobiont phenome and (**B**) *S. siderea* host only gene expression clustered by Symbiodiniaceae genus (0% *C. goreaui* in light blue to 100% *C. goreaui* in green as seen in the inset of **B**). Circles represent *C. goreaui*-hosting corals and triangles depict *D. trenchii* corals. Arrows represent significant (*P* < 0.05) correlation vectors for phenomic parameters (**A** only) and ellipses represent 95% confidence based on multivariate t-distributions.

To assess how experimental treatments influenced coral phenome, we conducted a PCA. Experimental *p*CO_2_ (*P* = 0.004) and temperature (*P* = 0.018) significantly altered the holobiont phenome (Fig. S2; Table S2), while Symbiodinacaeae genus (*P* = 0.28) and reef environment did not clearly affect holobiont phenome (*P* = 0.85; Fig. S2; Table S2). The first two PCs explained approximately 53% of the variance in *S. siderea* holobiont phenotypic responses (Fig. 3A). Variation along PC1 was driven by host carbohydrates,- protein content, algal symbiont chlorophyll content, and symbiont cell density. PC2 was driven by differences in hosted Symbiodinacaeae genus, host lipid content, chlorophyll a per cell, and color intensity. Overall, *C. goreaui*-hosting fragments exhibited faster calcification rates while *D. trenchii* fragments tended to have higher lipid content and chlorophyll a per symbiont cell (Fig. 3A).

### Coral host gene expression is driven by Symbiodiniaceae genus

**Fig. 4:**
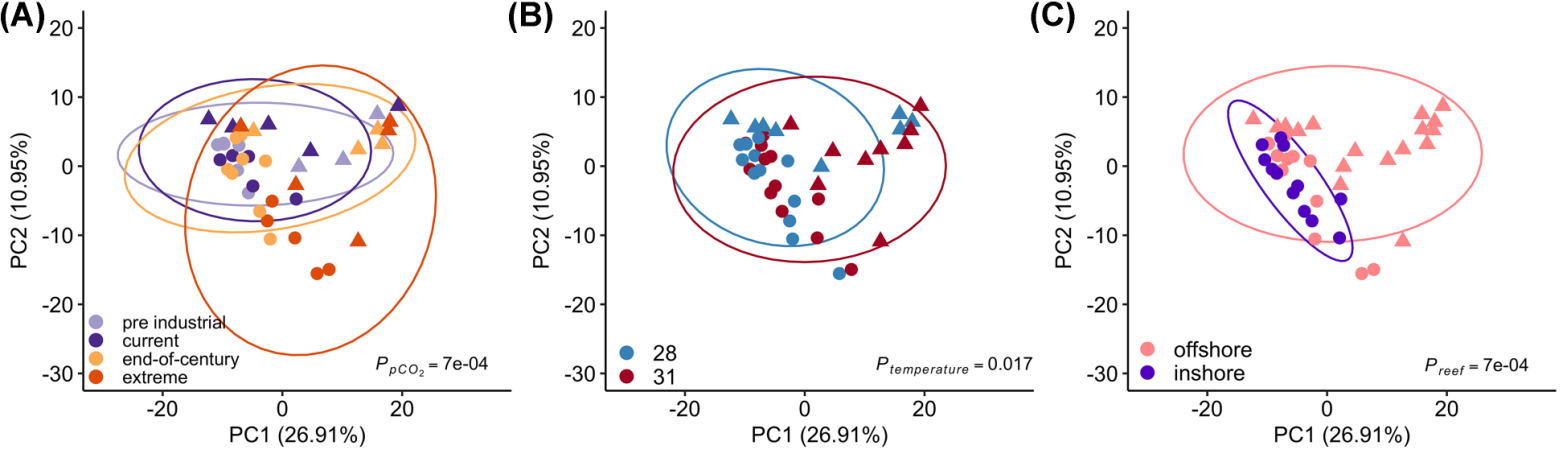
PCA of *S. siderea* host gene expression grouped by (**A**) *p*CO_2_ (280 μatm light purple; 400 μatm dark purple; 700 μatm light orange; 2800 μatm dark orange), (**B**) temperature treatment (28°C blue; 31°C red), and (**C**) reef environment (offshore pink; inshore purple). Ellipses represent 95% confidence based on multivariate t-distributions. Circles represent *C. goreaui*-hosting corals and triangles depict *D. trenchii* corals.

PCA of overall host gene expression profiles explained approximately 38% of the expression differences with two PCs (Fig. 3B). Host gene expression profiles were largely differentiated by which Symbiodiniaceae genus corals hosted (*P* = 0.001; Figs. 3B and 4) and host genotype itself (*P* = 0.001; Fig. S3). However, *p*CO_2_ treatment (*P* = 0.001), reef environment (*P* = 0.012), and temperature treatment (*P* = 0.017; Fig. 4; Table S3) were also significant, though their effects were less clear. Differential expression analysis identified few significant DEGs in response to treatment, though most of these were found in the extreme *p*CO_2_ treatment under warming (Fig. S4)

Gene expression profiles of *C. goreaui*-only reads were significantly influenced by *p*CO_2_ treatment (*P* = 0.03; Fig. S5A), resulting in 1,765 DEGs in the extreme *p*CO_2_ treatment under warming (Fig. S5B). Consistent with patterns observed in the *S. siderea*-only dataset, gene expression profiles of the coral holobiont (*S. siderea* + *C. goreaui + D. trenchii*) were significantly influenced by Symbiodiniaceae genus hosted (*P* = 0.001), reef environment (*P* = 0.001), *p*CO_2_ treatment (*P* = 0.002), and colony (*P* = 0.001; Fig. S5C). Again, only the extreme *p*CO_2_ treatment under warming drove significant DEGs in the holobiont (Fig. S5D).

### Discriminant analysis of principal components

**Fig. 5:**
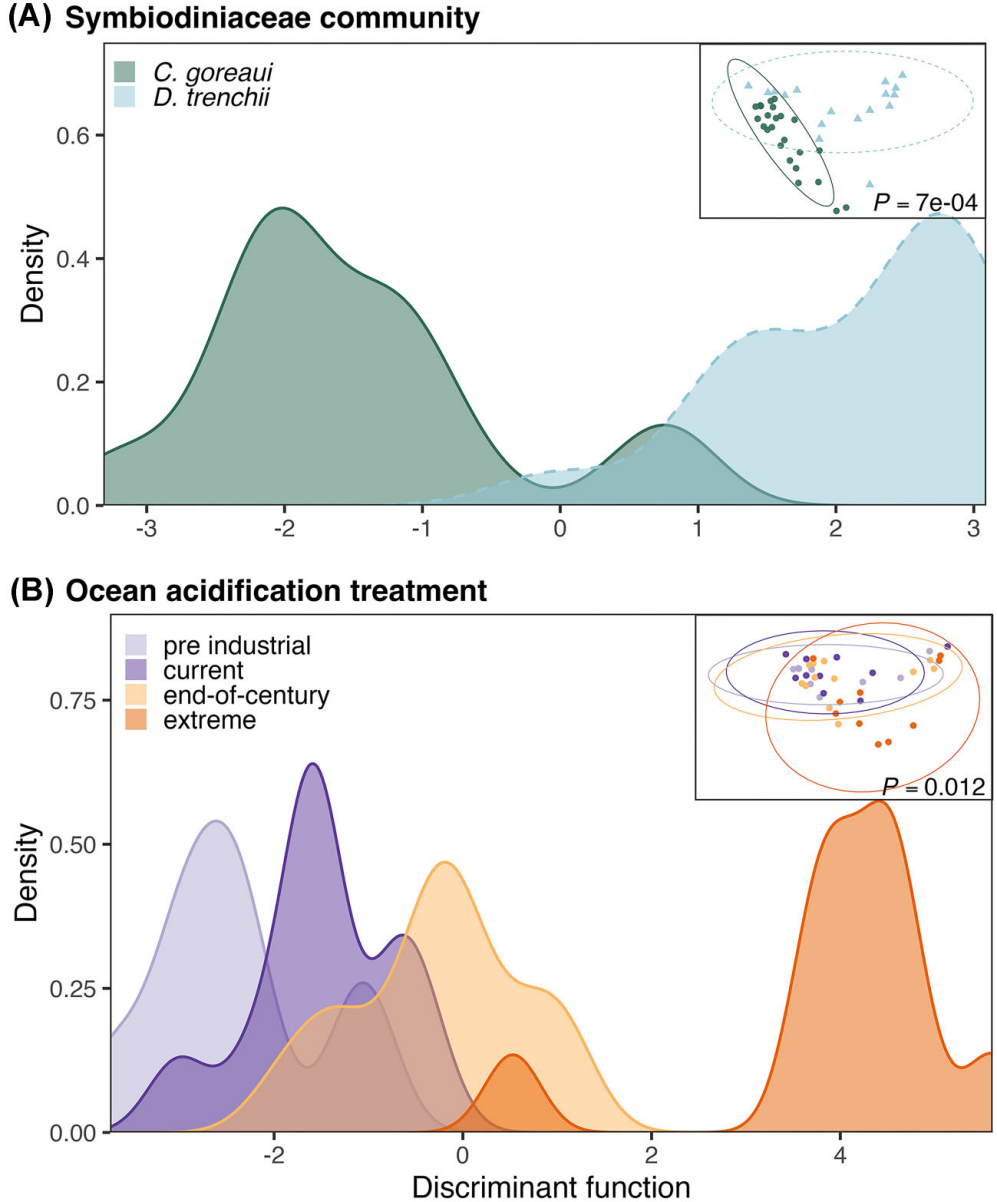
Density plot from the DAPC assessing effects of (**A**) Symbiodiniaceae genus (*Cladocopium goreaui*, dark blue; *D. trenchii*, light blue) and (**B**) *p*CO_2_ treatment (pre industrial, light purple; current day, dark purple; end-of-century, light orange; extreme, dark orange) on the coral host gene expression based on the first discriminant function. The PCA insert in the top right of each panel contains the PCA and significance of the coral host gene expression depicted by the corresponding parameter.

Results from host gene expression analyses were further supported by DAPC analyses, which identified different expression profiles between corals hosting different Symbiodiniaceae genera (Fig. 5A). Additionally, DAPC analyses highlighted differential host gene expression in samples reared in the extreme *p*CO_2_ treatment (pooling temperature treatments) compared to all other *p*CO_2_ treatments (Figs. 5B and S4A). A final DAPC to assess the overall effects of temperature (pooling *p*CO_2_ treatments) on host gene expression revealed overlapping expression profiles, suggesting that temperature had a minimal effect (Fig. S4B).

### Weighted gene correlation network analysis and GO enrichment

**Fig. 6:**
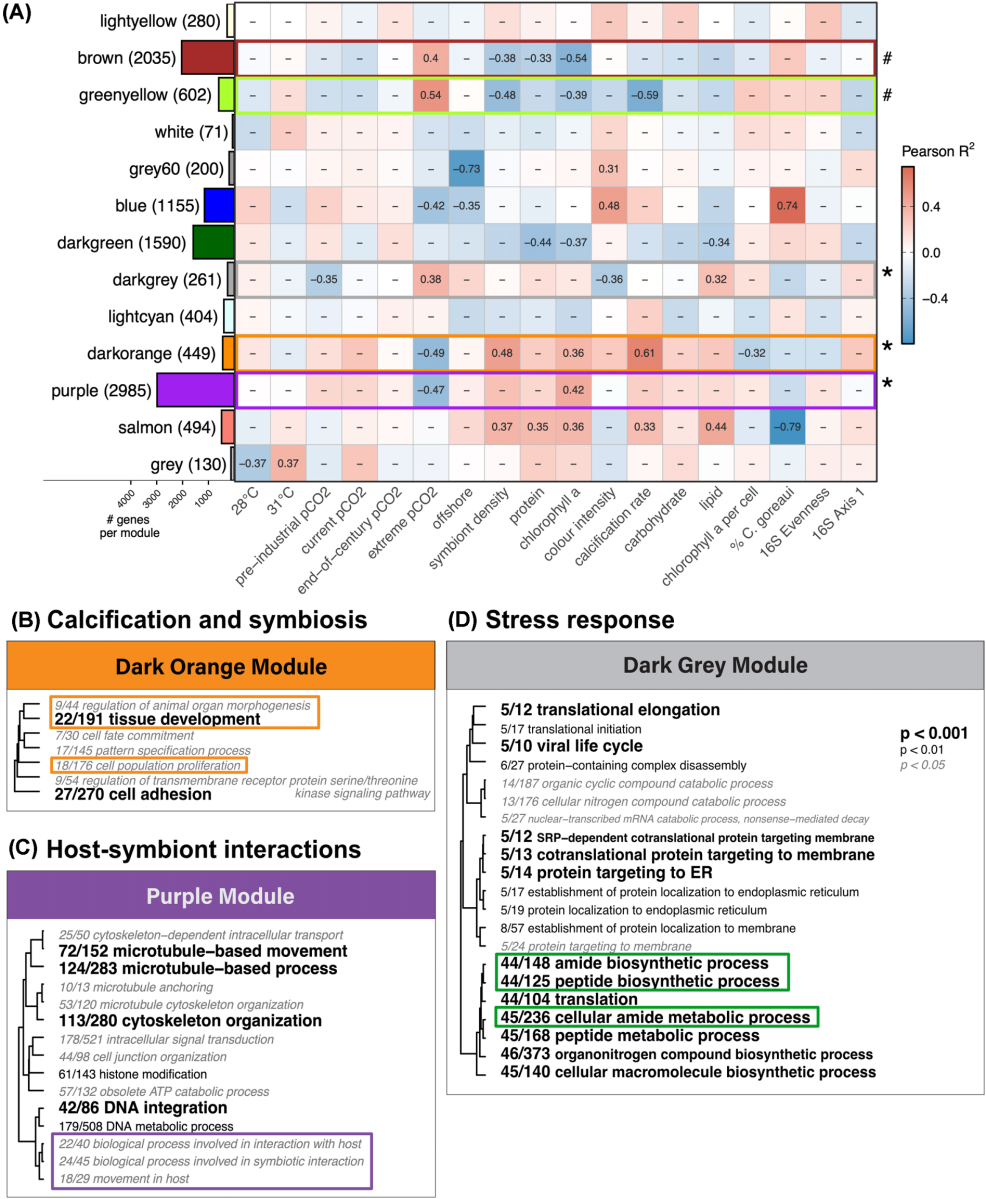
**A**) WGCNA heatmap (merging threshold of 0.65) showing correlations of gene modules (rows) with different phenotypic traits (columns). Significant correlations (alpha of *P* = 0.05) are denoted with the *R^2^* value of the correlation and the direction of the relationship is depicted by tile color (red = positive correlation; blue = negative correlation). Bars on the left of each row depict the number of genes within each module and the module color name. Modules with significant GO terms are identified by the corresponding color box around the row (“brown,” “greenyellow,” “purple,” “darkorange,” and “darkgrey”). The hash (#) denotes modules presented in the supplemental materials (Fig. S7). Significantly enriched GO terms from the Biological Process (BP) category of the (**B**) calcification and symbiosis module (“darkorange”), (**C**) host-symbiont interaction module (“purple”), and (**D**) the stress response module (“darkgrey”). Text size and boldness depict term significance (Fisher’s exact test) and terms highlighted by colored boxes are discussed further in the text. See Fig. S7 for GO enrichment results from all WGCNA modules, including “brown” and “greenyellow..”

WGCNA assigned 10,656 of the coral host *vst*-transformed isogroups to 13 modules (merging threshold of 0.65) (Fig. 6A). From these modules, GO enrichment analysis identified only five modules with significantly enriched GO terms (“brown,” “greenyellow,” “purple,” “darkorange,” “darkgrey”; Figs. 6 and S7-8). The “darkorange” module (449 genes) was positively associated with higher symbiont densities (*R*^2^ = 0.48), chlorophyll a content (*R*^2^ = 0.36), and faster calcification rates (*R*^2^ = 0.61), but negatively correlated with the extreme *p*CO_2_ treatment (*R*^2^ = −0.49). In general, this module was enriched for GO terms associated with skeletal growth, including *tissue development* (GO:0009888), and symbiosis, including *cell population proliferation* (GO:0008283) (Figs. 6A and S7). Therefore, we termed this the “calcification and symbiosis” module (Fig. 6A).

Genes in the “purple” module (2985 genes) were enriched with higher chlorophyll a content (*R*^2^ = 0.42), but depleted in the extreme *p*CO_2_ treatment (*R*^2^ = –0.47). This module was enriched for GO terms associated with symbiotic interactions, which included *movement in host* (GO:0044000) and *biological process involved in interaction with host* (GO: 0051701), thus we identified this module as the “host-symbiont interactions” module (Fig. 6A and C).

The “darkgrey” module (261 genes) was enriched in the extreme *p*CO_2_ treatment (*R*^2^ = 0.38) and with higher lipid content (*R*^2^ = 0.32) but depleted in the pre-industrial *p*CO_2_ treatment (*R*^2^ = –0.35) and with higher color intensity (*R*^2^ = –0.36). Several GO terms within this module (*amide biosynthetic process* [GO:0043604], *peptide biosynthetic process* [GO:0043043], *cellular amide metabolic process* [GO:0043603]) were previously identified by Dixon et al (2020) as a type B stress response, which led to this module being termed as the “stress response” module (Fig. 6A and C).

Both the “brown” (2035 genes) and “greenyellow” (602 genes) modules were positively associated with the extreme *p*CO_2_ treatment and negatively correlated with high algal symbiont (chlorophyll a, symbiont density) and host (protein in “brown”; calcification rate in “greenyellow”) physiological traits (Figs. 6A, S8). Enriched GO terms in the “greenyellow” module were associated with metabolism (i.e.*, oxoacid metabolic process* [GO:0043436], *sulfur compound metabolic process* [GO:0006790]) and homeostasis (i.e., *cellular homeostasis* [GO:0019725], *endoplasmic reticulum* [GO:0005793]). Similarly, the “brown” included GO terms associated with the environmental stress response (i.e.*, proton transmembrane transporter* [GO:0015078], *oxidoreduction-driven active transmembrane transporter* [GO:0015453], *chaperone-mediated protein folding* [GO:0061077]; Fig. S8). Together, these modules represent additional coral host stress response modules.

Genes in the “blue” module (1155 genes) were enriched in corals with higher color intensity (*R*^2^ = 0.48) and in corals hosting increased *C. goreaui* (*R*^2^ = 0.74), but were depleted in offshore corals (*R*^2^ = –0.35) and in the extreme *p*CO_2_ treatment (*R*^2^ = –0.42). The “darkgreen” module (1590 genes) was negatively correlated with several traits, including protein content (*R*^2^ = –0.44), chlorophyll a (*R*^2^ = –0.37), and total lipid content (*R*^2^ = –0.34). While the “salmon” module (494 genes) was negatively correlated with corals hosting more *C. goreaui* (*R*^2^ = –0.79), these genes were enriched in corals with higher symbiont densities (*R*^2^ = 0.37), chlorophyll a content (*R*^2^ = 0.36), protein content (*R*^2^ = 0.35), lipid content (*R*^2^ = 0.44), and faster calcification rates (*R*^2^ = 0.33). Despite these strong module eigengene correlations, GO enrichment analysis did not identify any significant GO enrichment in these modules (Fig. 6A).

Finally, the “grey” module (130 genes) was positively associated with corals in the elevated temperature treatment (*R*^2^ = 0.37) and negatively correlated in corals under ambient temperatures (*R*^2^ = –0.37). No significant correlations of genes with phenotypic traits were identified in the “lightyellow” (280 genes), “white” (71 genes), or “lightcyan” (404 genes) modules (Fig. 6A).

## Discussion

In a time of rapid environmental change, understanding the mechanisms underlying reef-building coral resilience is paramount. Variation in susceptibility across species and individuals within a species has been attributed to environmental history (Schoepf et al. 2022), host phenotype (Drury 2020), symbiont communities (Manzello et al. 2019; Ziegler et al. 2019), and the interactions between these factors (Fuller et al. 2020; Aichelman et al. 2025). Here, we demonstrate that coral-associated algal symbiont and microbiome communities remain unchanged under a variety of long-term (95-day) global change scenarios in the lab. Surprisingly, hosting different genera of Symbiodiniaceae algae had the strongest influence on coral gene expression patterns and the Symbiodiniaceae genus a coral fragment hosted was the best predictor of host gene expression, even after being reared for 95 days under vastly different ocean acidification and warming scenarios. While this association of host gene expression with different algal symbiont genera has been demonstrated previously in field-collected specimens (Barfield et al. 2018; Helmkampf et al. 2019) and after short-term stress experiments (DeSalvo et al. 2010; Cunning and Baker 2020; Connelly et al. 2023), our results demonstrate that this association drives gene expression patterns even after prolonged exposure to significant environmental stressors, suggesting that algal symbiont community composition drives some functional changes in corals.

### Microbial community composition shows limited shifts in response to global change

Significant progress has been made in understanding how various coral holobiont partnerships impact phenotypes, particularly under global change (Bourne et al. 2016; Bove et al. 2022b; Maire et al. 2022). For example, coral-associated algal communities have been observed to shift to the more putatively thermal tolerant *D. trenchii* following thermal stress events (Cunning, Silverstein and Baker 2015b; Silverstein et al. 2015; Thomas et al. 2019; Quigley et al. 2022). This algal symbiont shuffling is thought to provide fitness benefits to the coral host, including increased bleaching resistance (Cunning et al. 2015b), shifts in photochemical efficiency (Cunning et al. 2018), and improved metabolic performance (Ros et al. 2021). Despite previous work identifying algal symbiont shifts from *Cladocopium* to *Durusdinium* in *S. siderea* under extreme thermal stress (Davies et al. 2018), we observed no consistent shifts in algal symbiont community in response to our acidification and/or warming treatments. In fact, three of the six *S. siderea* colonies exclusively hosted *C. goreaui*, regardless of experimental treatment, suggesting these colonies may have lacked the necessary symbiont diversity required to shuffle. Of the remaining three colonies that hosted mixed *C. goreaui* and *D. trenchii* communities, there was notable within-colony variation across fragments. However, no clear shifts in response to temperature or acidification treatments were observed. Interestingly, only corals collected from the offshore site hosted *D. trenchii*, despite its more classic association with inshore environments (Garren et al. 2006; Stat and Gates 2011; Turnham et al. 2025). Previous work has identified similar Symbiodiniaceae diversity patterns in field-sampled *S. siderea* corals in southern Belize (Baumann et al. 2018), suggesting that these algal community patterns are common at these sites.

Although algal community shifts were not observed in response to treatments, it is possible that functional variation within each genus exists. Algal symbiont gene expression was only minimally explored here due to the presence of multiple genera. However, limited algal gene expression responses in *S. siderea-*associated *C. goreaui* collected from these same reefs were previously observed after 95 days under a variety of ocean acidification conditions (Davies et al. 2018). In addition, when *S. siderea* colonies from a similar region of Belize were reciprocally transplanted across environments for 3.5, no shifts in algal gene expression responses were observed (Castillo et al. 2024). Instead, in both cases, constitutive functional variation associated with reef environments was observed. While differences in algal communities were unexpected based on previous work showing that all pigmented *S. siderea* from these same sites were dominated by *C. goreaui* (Davies et al. 2018), our replication across factors was limited, making evaluations of within-genera symbiont gene expression challenging. Thus, future work exploring how coral hosts associating with different genera from the same reef environment respond to global change stressors represents a promising area of future research (Bove et al. 2022).

Similar to Symbiodiniaceae diversity, coral bacterial communities have been observed to change after exposure to ocean acidification and warming (Grottoli et al. 2018; Ziegler et al. 2019; Li et al. 2023), and have also been associated with different Symbiodiniaceae genera (Matthews et al. 2020). Even though we observed differences in Symbiodiniaceae community composition, we did not detect differences in bacterial diversity across coral fragments. This is surprising given that *S. siderea* are known to host distinct bacterial communities when collected from different habitats across Belize (Speare et al. 2020). However, these corals were maintained under experimental conditions for approximately six months, so it is possible that the observed bacterial communities may be due to their convergence under common garden mesocosm conditions (Galand et al. 2018). Alternatively, it is possible that *S. siderea* maintains relatively stable bacterial communities regardless of experimental conditions. Corals that do not modify their microbiome in response to environmental change (*i.e*., microbiome regulators) may exhibit higher host plasticity as a mechanism for resilience (Voolstra and Ziegler 2020). Thus, stable bacterial communities coupled with broad physiological responses observed in *S. siderea* hosts may support the hypothesis that this species remains ubiquitous on Caribbean coral reefs due to its capacity for either plasticity (Castillo et al. 2024) or resistance (Banks and Foster 2017 ) under environmental stress. Here, plasticity may manifest as constitutive robustness rather than shifts in holobiont composition, suggesting that *S. siderea* may rely on stable symbiotic partnerships and its broad capacity for physiological buffering to resist changing environmental conditions. However, caution should be taken in interpreting these patterns because there is also the potential that these corals simply acclimated to the common garden aquarium conditions during the experiment since initial microbial community compositions *in situ* were not assessed.

Although bacterial diversity did not differ across treatments, bacterial community compositions shifted at the class level in response to environmental drivers. Temperature was the primary structuring factor, with several classes associated with stable coral microbiomes (e.g., Actinobacteria, Gammaproteobacteria, Planctomycetes) declining under elevated temperature. These taxa are commonly implicated in core holobiont functions, including nutrient cycling and microbial homeostasis (Bourne et al. 2016; van Oppen and Blackall 2019). In contrast, taxa associated with anaerobic metabolism increased under elevated temperature, including members of Desulfobacterota and Clostridia, consistent with shifts previously observed in thermally stressed corals (Vega Thurber et al. 2009; Bourne et al. 2016). This shift toward anaerobic-associated taxa is consistent with a stress-associated restructuring of the coral microbiome under heat stress (Vega Thurber et al. 2009; Peixoto et al. 2017 ). Symbiont identity also influenced bacterial community composition with corals hosting *D. trenchii* exhibiting enrichment of several taxa relative to corals hosting *C. goreaui*, including Nitrospinia. Nitrospinia are nitrite-oxidizing bacteria involved in nitrification, suggesting potential effects of symbiont identity on nitrogen cycling within the holobiont (Wegley et al. 2007; Matthews et al. 2020). Finally, ocean acidification did not significantly affect bacterial community composition, indicating limited microbial restructuring under this stressor over the experimental period, consistent with prior work showing stronger microbial sensitivity to thermal stress than acidification (Ziegler et al. 2017).

### Algal symbiont community composition, not warming and/or acidification, controls host gene expression

Coral-symbiont interactions play a critical role in the long-term success of tropical reef-building corals (Muscatine and Porter 1977; Ros et al. 2021). Here, we show that the Symbiodiniaceae genera a coral hosts is the strongest predictor of host gene expression, regardless of experimental conditions. While previous work has demonstrated strong connections between coral host phenotype and Symbiodiniaceae genus (Cunning et al. 2015a; Hoadley et al. 2021; Allen-Waller and Barott 2023), our results highlight that Symbiodiniaceae associations shape functional variation in host gene expression.

This pattern of symbiont-associated host gene expression is not novel. In fact, Barfield et al. (2018) observed that corals from the same environment hosting different algal genera exhibited functional variation in gene expression. Cunning and Baker (2020) went one step further and showed that when corals hosting *Cladocopium* shuffled their symbionts to host *Durusdinium*, strong differences in expression were observed with *Durusdinium*-shifted corals exhibiting gene expression profiles more similar to those observed in *Cladocopium*-hosting corals under heat challenge. It is also possible that what we perceive as transcriptome-wide modifications of host gene expression are simply the result of different Symbiodiniaceae strains modulating gene expression patterns of gastrodermal cells, which are the most numerous cells in cnidarians and can show strong functional variation based on symbiosis status (Valadez-Ingersoll et al. 2025). However, this pattern may also be the result of divergent energetic demands associated with hosting different Symbiodiniaceae genera. For example, it has been shown that corals hosting *Durusdinium* spp. may receive proportionally less photosynthate than conspecifics hosting other genera (Pettay et al. 2015; Allen-Waller and Barott 2023). These changes in energetic requirements may in turn shift patterns of host gene expression.

Interestingly, we only identified one WGCNA module associated with hosting different Symbiodiniaceae genera. However, no significant enrichment was detected in this module, suggesting that hosting different Symbiodiniaceae genera leads to broad changes in host gene expression, but not clear functional enrichment. This is surprising given that our results, and those from previous studies (Matthews et al. 2018; Wang et al. 2024), demonstrate distinct functional holobiont responses based on Symbiodiniaceae associations. However, our data only explore host gene expression and we did not characterize functional changes in algal symbionts that may contribute to changes in the holobiont. Thus, we urge future work to consider the unique contributions of both the coral host and algal symbionts by specifically targeting the molecular and physiological contributions of each partner to holobiont phenomes.

Although coral host gene expression was clearly driven by Symbiodiniaceae genus, the effect of algal symbiont genus on holobiont physiology was less clear. In fact, Symbiodiniaceae genus was not a significant predictor of variation in holobiont physiology in this experiment. While previous research has demonstrated differences in holobiont physiology associated with Symbiodiniaceae genus (Jones and Berkelmans 2010; Cooper et al. 2011; Matthews et al. 2018; Hoadley et al. 2019), these patterns were more subtle in our data. For example, corals hosting *D. trenchii* tended to have higher lipid stores at the end of the experimental period, suggesting that hosting *D. trenchii* may contribute to the host investing in more long-term energy stores. This pattern has been observed previously and is hypothesized to be a mechanism for energy storage in the event of algal symbiont loss, particularly due to thermal bleaching (Cooper et al. 2011). However, it is important to note that only a single host genotype was dominated by *D. trenchii*, limiting our host-symbiont sample size. Alternatively, these higher energy stores may represent a metabolic trade-off in the coral resulting in lower calcification rates compared to corals hosting *C. goreaui* (Jones and Berkelmans 2010; Ortiz et al. 2013; Cunning et al. 2015a). Indeed, corals hosting *D. trenchii* were associated with lower calcification rates than those hosting *C. goreaui*, suggesting that *S. siderea* may employ different growth strategies based on the algal symbionts they host. While this strategy may preserve corals in the near term, chronic reduced calcification rates will likely result in detrimental declines in coral cover across Caribbean reefs. Regardless, as we continue to see more frequent and severe warming events happening across the Caribbean (Muñiz-Castillo et al. 2019; Bove et al. 2022c; Birkart and Álvarez-Filip 2025) and globally (Oliver et al. 2018; Goreau and Hayes 2024), coral species that are able to employ different metabolic strategies under environmental change may have the best success at populating future reefs.

### Warming and acidification lead to functional changes in host gene expression

Understanding the molecular mechanisms underlying phenotypes under changing ocean conditions is important for predicting future reef communities. For example, coral calcification is closely linked to its partnership with algal symbionts (Gattuso et al. 1999; Allemand et al. 2004), benefiting from the photosynthetic byproducts and modifications to calcifying fluid chemistry that support increased metabolism and growth. Here, we see further evidence supporting this relationship through the calcification module (“darkorange module”), which highlights the close relationship between coral calcification rates and symbiont physiology. Within this module, we identified enrichment of pathways associated with growth and movement of ions across membranes, which were positively correlated with faster calcification rates, higher symbiont densities, and increased chlorophyll a content. Interestingly, we see an inverse relationship between the eigengene expression of the calcification module and corals reared in the most extreme ocean acidification and warming treatment, suggesting these extreme conditions inhibited coral growth. Indeed, ion transport and ion channel pathways are known to be responsive to changing seawater pH conditions, generally being upregulated under moderate acidification stress (Vidal-Dupiol et al. 2013; Davies et al. 2016; Bernardet et al. 2019; Glazier et al. 2020). However, here we observed downregulation of these pathways coupled with reduced growth rates, indicating that these experimental conditions (warming and acidification) likely reduce the capacity of *S. siderea* to maintain net calcification. This downregulation has been previously reported and attributed to the combination of temperature treatment, acidification conditions, and exposure duration used (Kaniewska et al. 2015; Glazier et al. 2020). While warming alone did not elicit a strong gene expression response in our study, previous work found that *S. siderea* exhibited transcriptomic signatures of disrupted homeostasis under slightly higher levels of thermal stress (32°C vs. 31°C in this study) (Davies et al. 2016). Davies et al. (2016) also identified gene expression patterns consistent with increased respiration from corals under end-of-century (604 μatm) and extreme (2553 μatm) *p*CO_2_ conditions, suggesting increased energetic demands on the coral to maintain growth under ocean acidification conditions. These findings contrast our results where we only observed *S. siderea* elicit a stress response signature under the combined effects of ocean acidification and warming. Overall, these results highlight that severity of stressor, the ways in which stressors interact, and stressor duration all have a strong impact on coral responses to environmental stress (McLachlan et al. 2020).

Similar to the calcification module, the “purple” module (termed here the host-symbiont interaction module) was positively associated with symbiont density and chlorophyll *a* content. This module was enriched with GO terms associated with symbiosis, such as *movement in host* and *biological processes involved in symbiotic interaction*. The enrichment of symbiosis-related GO terms in corals with enhanced symbiont physiology highlights potential mechanisms by which the coral hosts may support associations with Symbiodiniaceae. Symbiosis regulation in coral holobionts likely results from changes in nutrient cycling between partners (Muscatine and Porter 1977; Rädecker et al. 2015; Valadez-Ingersoll et al. 2025) with coral hosts controlling nitrogen availability to the algal symbionts, limiting their population growth while optimizing photosynthetically fixed carbon translocation (Lesser 2013; Rädecker et al. 2021). Notably, the host-symbiont interaction module was negatively correlated with exposure to extreme *p*CO_2_ and elevated temperature, indicating a possible functional shift in symbiosis under these stress conditions. This pattern may indicate early signs of symbiosis breakdown that were not identified in other physiological analyses. Rather, the response may result from changing nutrient exchange within the coral holobiont that favor greater nitrogen availability to the algal symbionts, leading to reduced carbon availability to the host, and ultimately destabilizing the symbiosis (Rädecker et al. 2021; Marangon et al. 2025).

The “darkgrey” module—referred to here as the stress response module—showed enrichment of GO terms related to translation and protein synthesis, and this module was positively correlated with the most extreme *p*CO_2_ and temperature conditions. Such enrichment is consistent with typical environmental stress responses (ESR), which often involve upregulation of protein synthesis machinery to produce stress-related proteins (Desalvo et al. 2008; López-Maury et al. 2008; Rosic et al. 2014). Further, increased expression of ribosomal genes has been linked to thermal stress (Kenkel and Matz 2016) and may serve as an early indicator of bleaching (Desalvo et al. 2008; Dilworth et al. 2024). Although no visual bleaching was observed, the inverse relationship between this stress module and coral color intensity suggests the potential onset of bleaching. Interestingly, several of the enriched terms identified in this module were also reported by Dixon et al. (2020) in their meta-analysis of the Type B ESR in corals exposed to milder stress conditions. Collectively, these findings indicate that the corals in this study were mounting a moderate stress response under even the most extreme experimental conditions, reinforcing the idea that *S. siderea* can maintain growth across a range of environmental stressors (Baumann et al. 2016; Okazaki et al. 2017; Bove et al. 2019), and may continue to persist on Caribbean reefs despite ongoing ocean change.

## Conclusions

Overall, our study reveals that the Caribbean coral *S. siderea* shows remarkable resilience under projected ocean acidification and warming conditions, exhibiting only moderate gene expression responses after three months of exposure to even the most extreme conditions. While we did observe reduced growth rates and downregulation of calcification-related genes under extreme acidification and warming scenarios, these conditions are unlikely to be experienced on these reefs in Belize within the next century. Notably, Symbiodiniaceae community composition was not significantly altered across experimental treatments, yet, algal symbiont genus served as the primary driver of host gene expression patterns. Physiologically, *Durusdinium* coral hosts exhibited higher lipid content but reduced calcification rates compared to those assigned as *Cladocopium* hosts, suggesting different metabolic strategies based on symbiont genera. These findings suggest that future reef persistence likely depends on the combination of short-term plastic phenomic and gene expression responses along with metabolic strategies conferred by specific Symbiodiniaceae partnerships. Understanding how symbiont-mediated functional trade-offs influence growth, energy storage, and stress tolerance is essential for predicting reef trajectories under changing oceanic conditions.

## Supplementary Material

icag062_Supplemental_Files

## Data Availability

Raw sequences for all gene expression samples and ITS2/16S metabarcoding are available on the NCBI Short Read Archive (SRA) under BioProject number PRJNA1465334. All other data and code can be accessed at GitHub (https://github.com/seabove7/SSID-gene-expression) and Zenodo (https://doi.org/10.5281/zenodo.20448169).
