# Supplementary material for: Strong Symbiodiniaceae Influence on Coral Gene Expression Under Ocean Acidification and Warming
Source: Integr Comp Biol. 2026 Jun 3;66:icag062. doi: 10.1093/icb/icag062 (PMC13321127; doi:10.1093/icb/icag062)
Supplement: icag062_Supplemental_Files [file icag062_supplemental_files.zip › icb-2026-0026-File009.pdf]

Supplemental Materials for manuscript: Strong Symbiodiniaceae influence on coral gene expression under ocean acidification and warming

Colleen B Bove[1,2,3*], Karl D Castillo[3,4], Annabel M Hughes[2,5], Justin B Ries[5], Sarah W Davies[2]

[1] Department of Biology, Ursinus College, Collegeville, Pennsylvania, USA
[2] The Department of Biology, Boston University, Boston, Massachusetts, USA
[3] Environment, Ecology, and Energy Program, The University of North Carolina at Chapel Hill, Chapel Hill, North Carolina, USA
[4] The Department of Marine Science, The University of North Carolina at Chapel Hill, Chapel Hill, North Carolina, USA
[5] Department of Marine and Environmental Sciences, Northeastern University, Nahant, MA, USA
[*]*Corresponding author: cbove@ursinus.edu*

Supplemental Figures

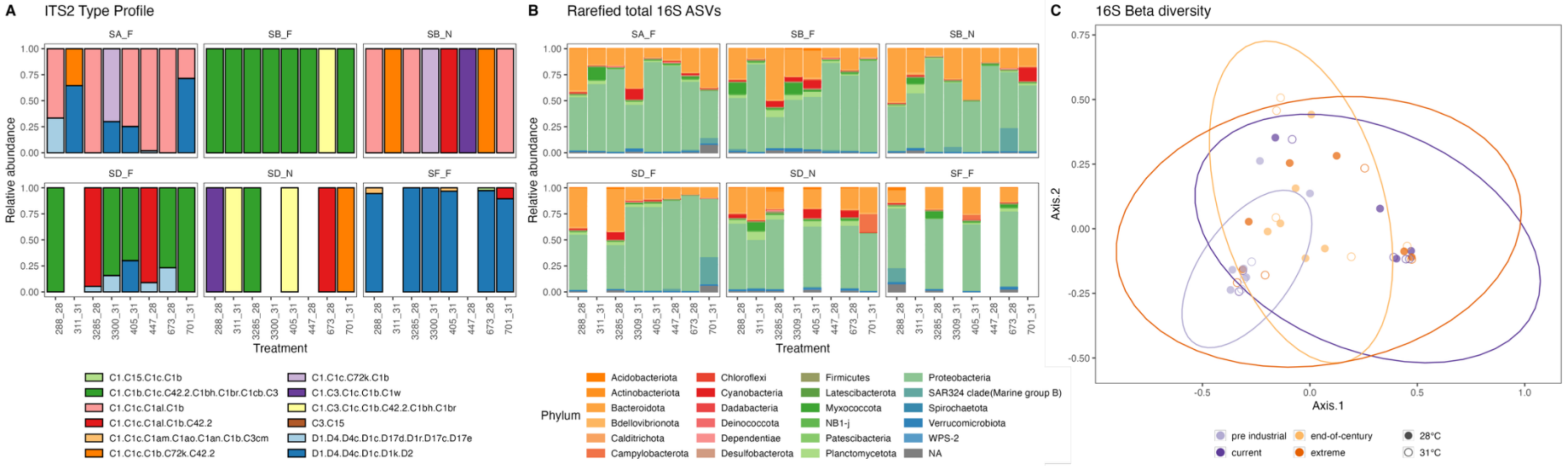

Figure S1. Algal (**A**) and bacterial community composition **(B**) of coral host samples grouped by colony with treatment on the x-axis. Relative algal community compositions are shown at the ITS2 Type Profile level, and these profiles were collapsed into dominant ITS2 types for all downstream analyses (**Figure 1B**). Relative bacterial compositions are shown here at the phylum level. Overall bacterial beta diversity (**C**) coloured by pCO_2 treatment (pre industrial, light purple; current day, dark purple; end-of-century, light orange; extreme, dark orange) with temperature treatments represented by symbols (28°C solid circles; 31°C open circles).

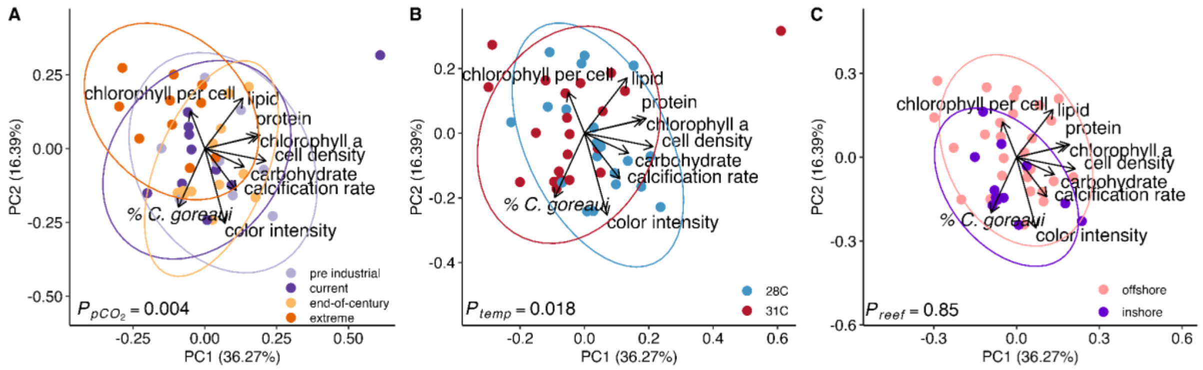

Figure S2. Principal component analyses of *Siderastrea siderea* coral holobiont phenomes depicted by (**A**) pCO_2 (280 µatm light purple; 400 µatm dark purple; 700 µatm light orange; 2800 µatm dark orange), (**B**) temperature treatment (28°C blue; 31°C red), and (**C**) reef environment (offshore pink; inshore purple). Arrows represent significant ($p < 0.05$) correlation vectors for physiological parameters and ellipses represent 95% confidence based on multivariate t-distributions.

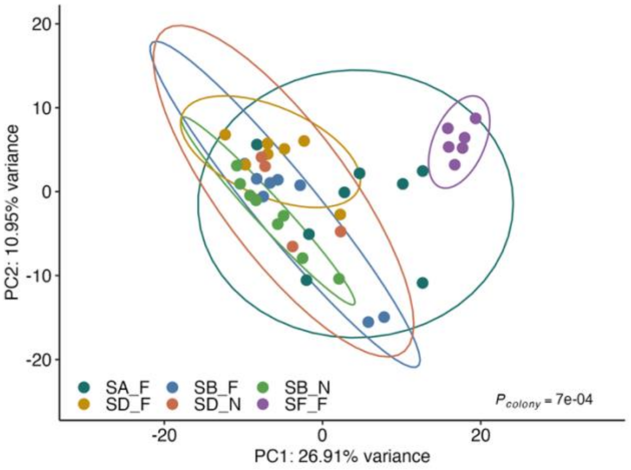

Figure S3. Principal component analysis of *Siderastrea siderea* host gene expression clustered and coloured by coral colony. Ellipses represent 95% confidence based on multivariate t-distributions.

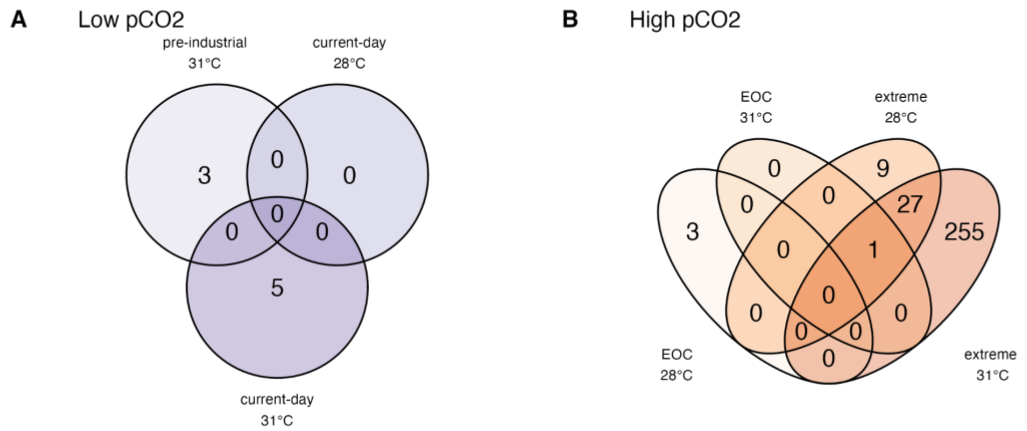

Figure S4. Venn diagrams of significantly differentially expressed genes (DEGs; Padj=0.05) identified in the (**A**) low pCO_2 treatments and (**B**) high pCO_2 treatments compared to the pre-industrial pCO_2 treatment at 28°C. No DEGs were shared between the low and high pCO_2 treatments and were therefore separated for visibility.

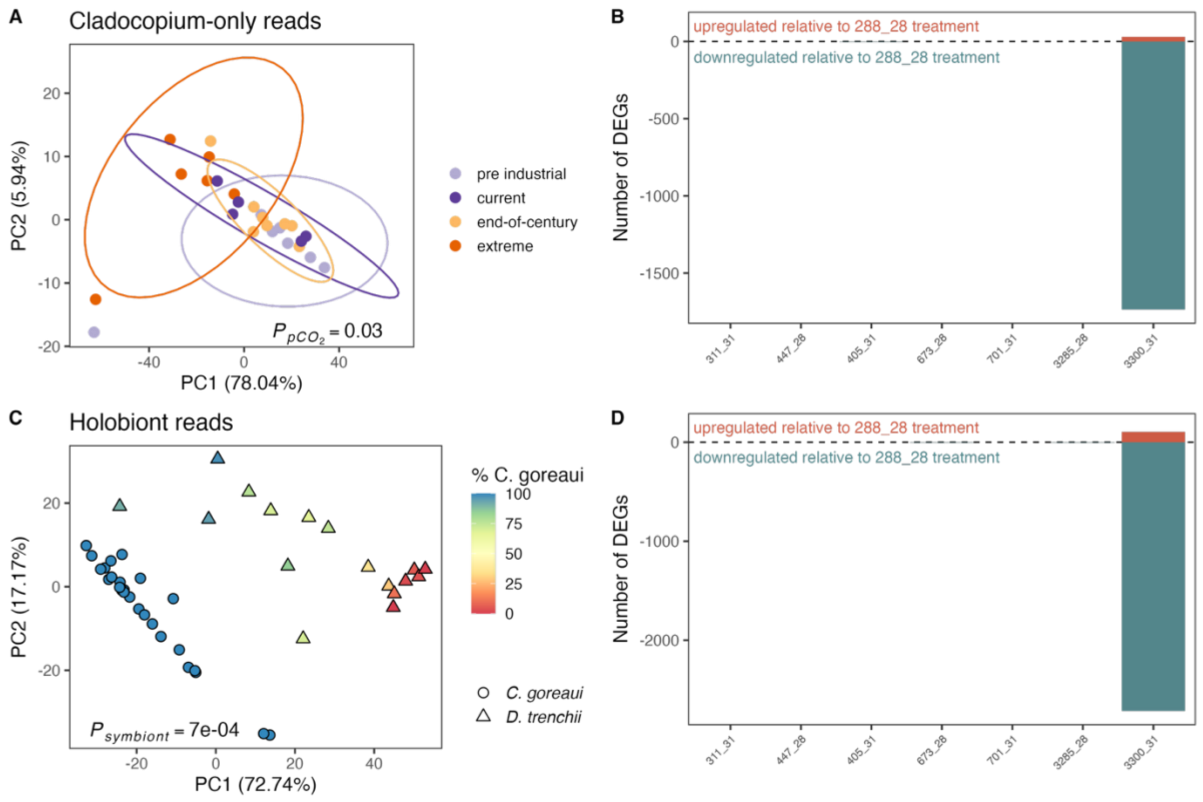

Figure S5. Overall gene expression patterns of (**A**, **B**) *C. goreaui* reads only or (**C**, **D**) coral holobiont (*S. siderea* + *C. goreaui* + *D. trenchii*) reads. Principal component analyses were performed on (**A**) *C. goreaui*-only reads depicted by pCO_2 treatment (280 µatm light purple; 400 µatm dark purple; 700 µatm light orange; 2800 µatm dark orange), and (**C**) holobiont reads depicted by % *C. goreaui* hosted. Significantly differentially expressed genes (DEGs; Padj=0.05) identified across treatments for (**B**) *C. goreaui*-only reads and (**D**) holobiont reads, with red representing upregulated genes and downregulated genes in blue compared to the pre industrial pCO_2 at 28°C treatment.

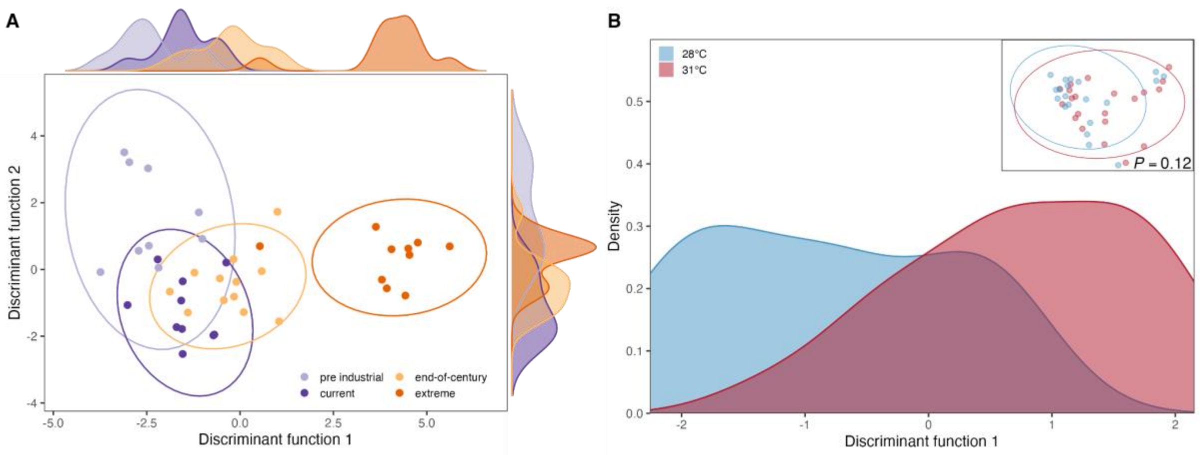

Figure S6. Density plot from the discriminant analysis of principal components (DAPC) assessing effects of (**A**) pCO_2 treatment (pre industrial, light purple; current day, dark purple; end-of-century, light orange; extreme, dark orange) and (**B**) temperature (28°C blue; 31°C red) on coral host gene expression. Panel **A** depicts the first two discriminant functions of the coral host gene expression based on pCO_2 treatment, while panel **B** represents a single discriminant function of temperature treatment.

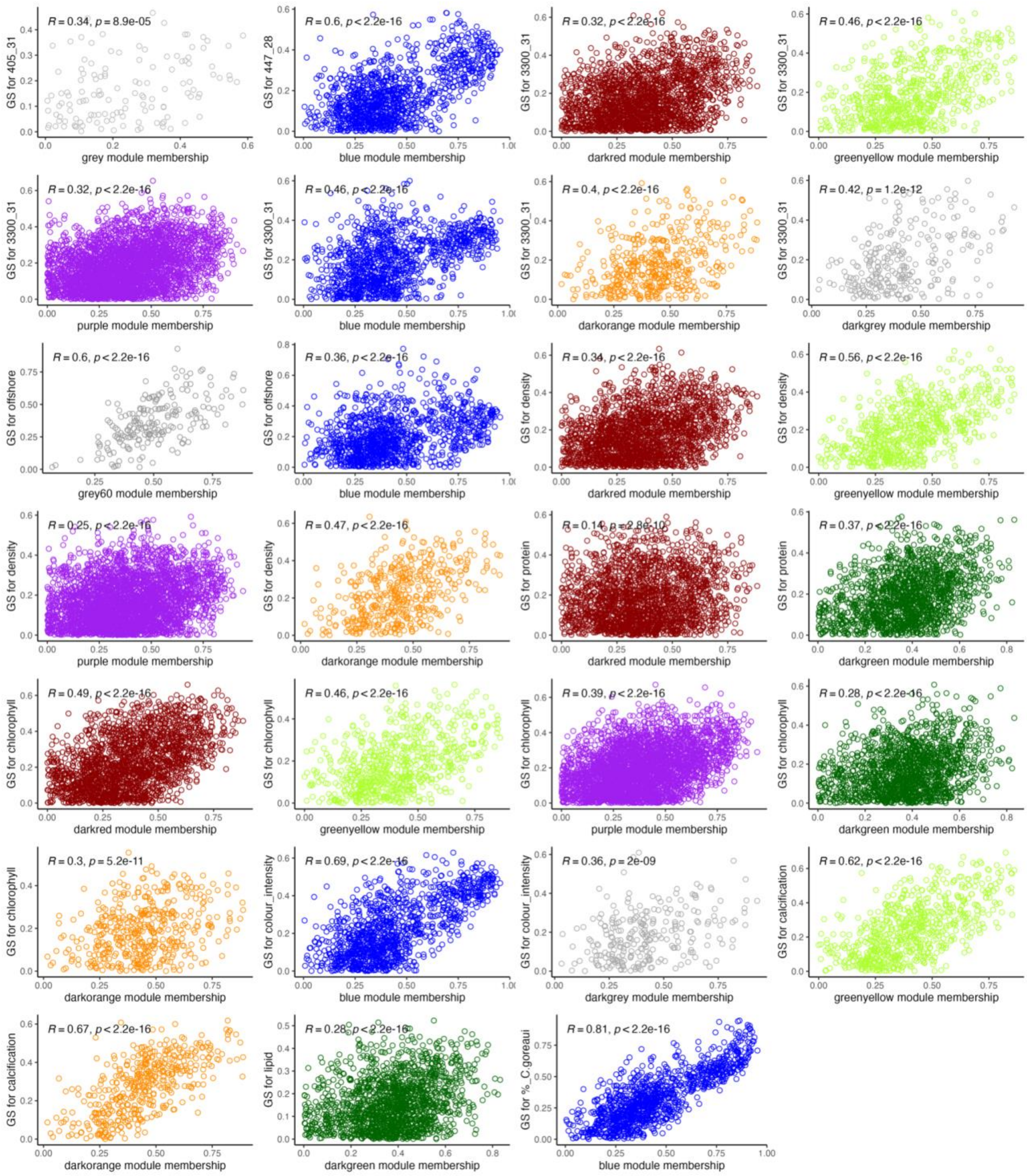

Figure S7. All identified WGCNA modules (from **Figure 6A**) correlated against significant traits with R^2 and p-values.

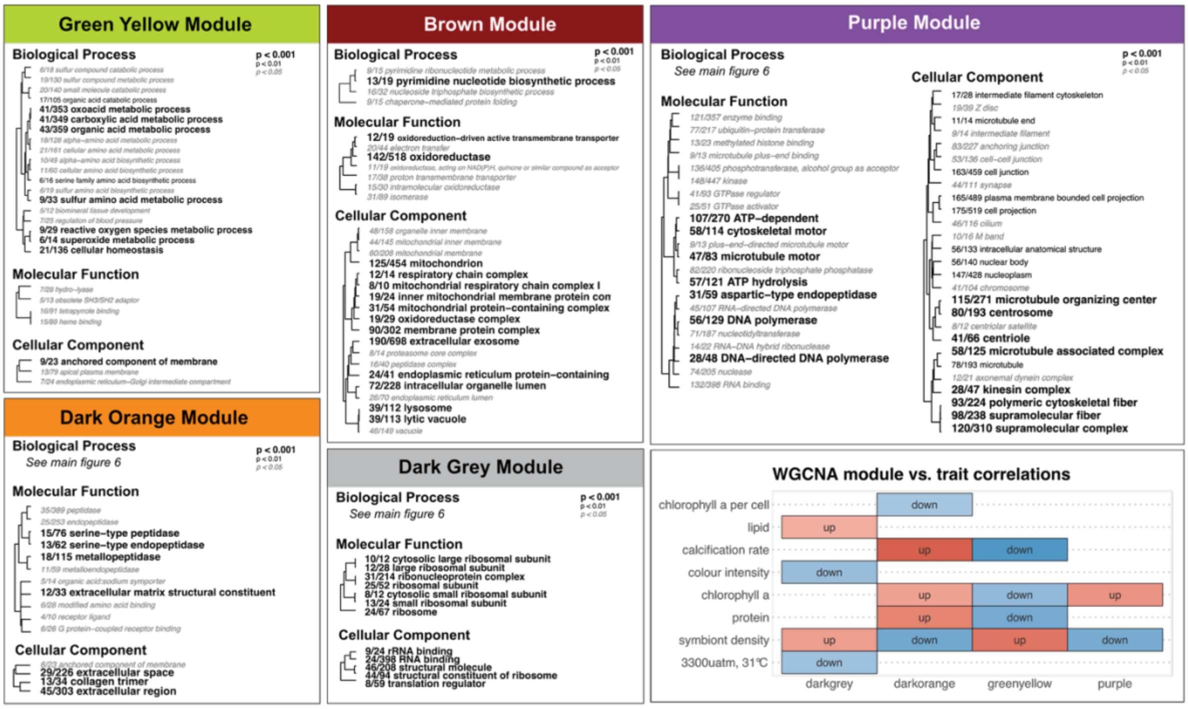

Figure S8. Significantly enriched Gene Ontology (GO) terms identified within WGCNA modules that correlate with different phenotypic traits of the coral seen in **Figure 6** in the main text. Text size and boldness depict term significance (Fisher's exact test). A simplified WGCNA heatmap showing correlations of gene modules (columns) with different phenotypic traits (rows) depicts significant correlations (alpha of p = 0.05), with tile colour representing the direction of the relationship (red = positive correlation; blue = negative correlation).

Supplemental Tables

Table S1. Summary of RNA libraries, including sample ID, mapped reads to the holobiont, percent of reads mapping to *S. siderea*, percent of reads mapping to combined symbiont (*C. goreaui* and *D. trenchii*), percent of reads mapping specifically to *C. goreaui*, and percent of reads mapping specifically to *D. trenchii*. Samples marked with an asterisk (*) denote samples that were identified as outliers and removed from analyses.

sample	Raw reads	Cleaned reads	Holobiont reads	Host reads (%)	Symbiont reads (%)	*Cladocopium* reads (%)	*Durusdinium* reads (%)
CFSA10	4,260,308	833,251	272,062	82.23	20.33	16.55	3.78
CFSA14	6,547,614	1,233,833	560,908	71.77	30.10	8.98	21.13
CFSA1	7,221,322	1,372,466	587,186	74.35	27.42	3.96	23.46
CFSA2	6,573,780	1,217,501	608,478	58.87	43.38	29.49	13.89
CFSA3	2,951,480	537,645	281,030	72.18	29.66	7.03	22.63
CFSA5	8,082,229	1,565,931	656,975	72.77	28.90	3.30	25.59
CFSA6	4,616,244	853,284	67,338	75.57	27.98	24.34	3.64
CFSA7	6,301,213	1,170,625	440,696	90.75	11.16	4.27	6.89
CFSB10	7,319,277	1,484,806	516,244	73.59	29.78	26.51	3.28
CFSB13	7,998,839	1,456,292	698,284	68.04	34.78	32.21	2.57
CFSB1	5,215,060	956,006	452,257	61.99	40.78	37.76	3.02
CFSB2	3,170,552	626,326	192,613	70.60	32.71	28.59	4.12
CFSB3	3,557,941	684,528	239,495	89.03	13.92	11.81	2.11
CFSB5	8,846,843	1,657,686	681,970	72.18	30.96	28.14	2.82
CFSB6	9,954,497	1,944,921	727,086	80.65	22.24	19.56	2.68
CFSB7	7,954,980	1,460,452	407,945	91.88	10.44	7.92	2.52
CFSD11	4,320,559	789,454	290,043	66.79	36.43	33.21	3.21
CFSD3	7,512,393	1,514,526	404,696	68.20	34.09	14.83	19.26
CFSD5	13,562,193	2,498,088	1,056,204	74.18	28.68	26.19	2.49
CFSD6	4,740,170	876,336	446,389	67.24	35.10	19.11	15.99
CFSD7	10,629,310	2,176,318	337,047	72.38	30.82	11.92	18.90
CFSD8	6,086,620	1,156,683	535,250	71.11	31.44	23.02	8.41
CFSD9	7,266,240	1,395,866	622,835	57.97	45.00	41.02	3.98
CFSF16	12,948,817	2,427,333	922,978	77.77	23.95	2.83	21.12

sample	Raw reads	Cleaned reads	Holobiont reads	Host reads (%)	Symbiont reads (%)	*Cladocopium* reads (%)	*Durusdinium* reads (%)
CFSF2	5,616,645	1,055,171	448,479	67.53	34.00	2.98	31.02
CFSF3	6,052,344	1,175,134	167,802	62.62	38.71	2.86	35.85
CFSF5	10,451,449	2,227,446	574,796	71.45	30.30	3.53	26.77
CFSF6	4,040,296	756,492	381,381	68.98	32.67	3.23	29.45
CFSF7	6,112,070	1,049,947	412,307	61.39	40.17	3.35	36.82
CNSB1	8,449,965	1,547,730	547,259	76.26	26.43	23.79	2.64
CNSB20	4,992,974	933,536	68,202	82.30	20.48	11.78	8.70
CNSB2	9,666,254	1,775,987	988,381	62.27	40.26	38.18	2.08
CNSB3	6,401,999	1,369,285	404,777	80.21	22.94	19.72	3.22
CNSB5	8,370,916	1,556,897	630,001	68.16	34.64	31.79	2.85
CNSB6	5,336,246	1,061,014	420,551	72.61	30.30	27.37	2.93
CNSB7	6,696,640	1,228,239	614,526	67.39	35.36	32.42	2.94
CNSB8	4,499,537	852,782	287,810	84.75	18.36	15.59	2.78
CNSD1	9,971,174	1,971,976	937,281	70.68	32.09	29.81	2.28
CNSD2	16,502		960	71.25	32.81	14.06	18.75
CNSD3	8,089,678	1,568,278	561,294	89.52	12.88	10.63	2.25
CNSD5	7,342,020	1,353,684	714,986	76.80	25.81	23.60	2.20
CNSD6	7,903,730	1,532,167	558,761	72.51	30.40	27.35	3.05
CNSD8	17,257		915	68.20	35.96	16.72	19.23

Table S2. PERMANOVA model output from coral holobiont phenomes using the *adonis2* function with 1500 iterations.

	Df	Sum of Squares	R^2	F	P-value
symbiont genera	1	1,616,075,573,006.00	0.02	1.17	0.28
temperature	1.00	8,434,771,433,198.00	0.11	6.09	0.02
pCO_2	3.00	20,911,087,766,678.00	0.27	5.03	0.00
reef environment	1.00	50,270,361,015.00	0.00	0.04	0.85
Residual	*34.00*	*47,085,231,466,742.00*	*0.60*		
Total	*40.00*	*78,097,436,600,638.00*	*1.0*		

Table S3. PERMANOVA model output from coral host gene expression using the *adonis2* function with 1500 iterations.

	Df	Sum of Squares	R^2	F	P-value
temperature	1.00	403.00	0.03	1.48	0.10
symbiont genera	1.00	1,823.00	0.13	6.72	0.00
pCO_2	3.00	1,379.00	0.10	1.69	0.02
reef environment	1.00	840.00	0.06	3.09	0.00
Residual	*34.00*	*9,230.00*	*0.68*		
Total	*40.00*	*13,677.00*	*1.0*		

Table S4. Summary table of the total number of differentially expressed genes by each contrast level compared to the 'control' conditions of 288 µatm, 28°C and across coral colonies, with SAF as the 'control' coral.

Contrast	Total_DEGs
Coral: SBF	541
Coral: SBN	540
Coral: SDF	516
Coral: SDN	301
Coral: SFF	421
Treatment: 311 uatm; 31 C	3
Treatment: 3285 uatm; 28 C	37
Treatment: 3300 uatm; 31 C	283
Treatment: 405 uatm; 31 C	5
Treatment: 447 uatm; 28 C	0
Treatment: 673 uatm; 28 C	3
Treatment: 701 uatm; 31 C	1

Table S5. Total number of genes within each identified WGCNA module represented in **Figure 4.**

Module name	Number of genes
grey	130
salmon	494
purple	2,985
darkorange	449
lightcyan	404
darkgrey	261
darkgreen	1,590
blue	1,155
grey60	200
white	71
greenyellow	602
brown	2,035
lightyellow	280
