# Supplementary figures and images for: Strong Symbiodiniaceae Influence on Coral Gene Expression Under Ocean Acidification and Warming
Source: Integr Comp Biol. 2026 Jun 3;66:icag062. doi: 10.1093/icb/icag062 (PMC13321127; doi:10.1093/icb/icag062)

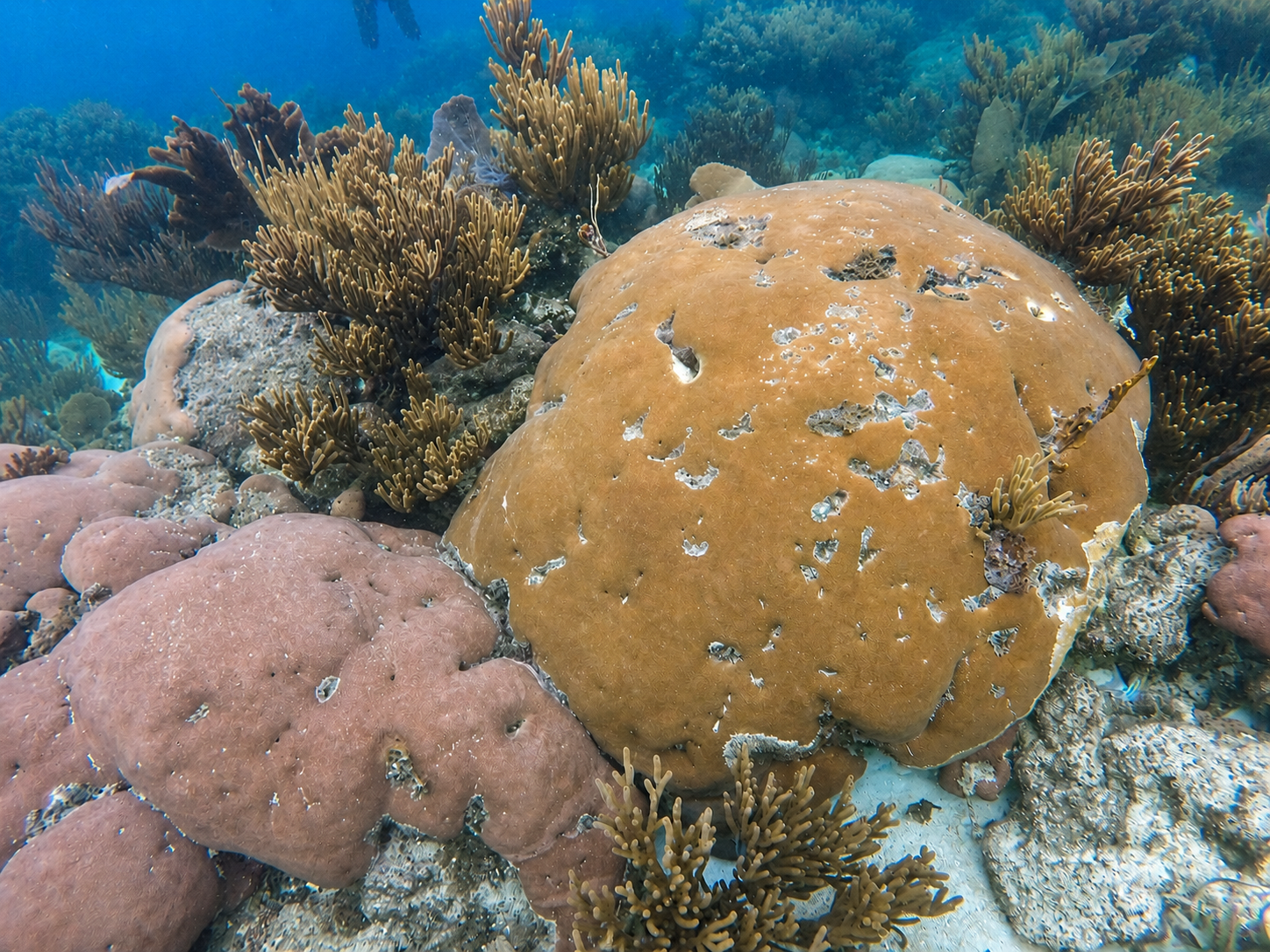

Supplement: icag062_Supplemental_Files [file icag062_supplemental_files.zip › icb-2026-0026-File001.png]
